# The *Escherichia coli* NarL receiver domain regulates transcription through promoter specific functions

**DOI:** 10.1186/s12866-015-0502-9

**Published:** 2015-08-26

**Authors:** Galit Katsir, Michael Jarvis, Martin Phillips, Zhongcai Ma, Robert P. Gunsalus

**Affiliations:** Department of Chemistry and Biochemistry, University of California, Los Angeles, USA; Department of Microbiology, Immunology, and Molecular Genetics, University of California, Los Angeles, USA; Molecular Biology Institute, University of California, Los Angeles, USA; Present address: San Jose City College, San Jose, CA USA; Present address: Ambry Genetics, Aliso Viejo, CA USA; Present address: NeuroInDx, Inc, Signal Hill, CA USA

## Abstract

**Background:**

The *Escherichia coli* response regulator NarL controls transcription of genes involved in nitrate respiration during anaerobiosis. NarL consists of two domains joined by a linker that wraps around the interdomain interface. Phosphorylation of the NarL N-terminal receiver domain (RD) releases the, otherwise sequestered, C-terminal output domain (OD) that subsequently binds specific DNA promoter sites to repress or activate gene expression. The aim of this study is to investigate the extent to which the NarL OD and RD function independently to regulate transcription, and the affect of the linker on OD function.

**Results:**

NarL OD constructs containing different linker segments were examined for their ability to repress *frdA-lacZ* or activate *narG-lacZ* reporter fusion genes. These *in vivo* expression assays revealed that the NarL OD, in the absence or presence of linker helix α6, constitutively repressed *frdA-lacZ* expression regardless of nitrate availability. However, the presence of the linker loop α5-α6 reversed this repression and also showed impaired DNA binding *in vitro*. The OD alone could not activate *narG-lacZ* expression; this activity required the presence of the NarL RD. A footprint assay demonstrated that the NarL OD only partially bound recognition sites at the *narG* promoter, and the binding affinity was increased by the presence of the phosphorylated RD. Analytical ultracentrifugation used to examine domain oligomerization showed that the NarL RD forms dimers in solution while the OD is monomeric.

**Conclusions:**

The NarL RD operates as an on-off switch to occlude or release the OD in a nitrate-responsive manner, but has additional roles to directly stimulate transcription at promoters for which the OD lacks independent function. One such role of the RD is to enhance the DNA binding affinity of the OD to target promoter sites. The data also imply that NarL phosphorylation results in RD dimerization and in the separation of the entire linker region from the OD.

**Electronic supplementary material:**

The online version of this article (doi:10.1186/s12866-015-0502-9) contains supplementary material, which is available to authorized users.

## Background

Bacteria, archaea, and lower eukaryotes rely on two-component signal transduction systems as a major strategy to monitor the extra- and intracellular environment for physical and biological changes [1]. These multi-protein phospho-relay signaling systems allow cellular adaptation to changes in nutrient availability, osmolarity, oxygen, redox potential, light, plus other cell viability and survival determining factors. The two-component paradigm involves phosphoryl transfer from a sensor kinase that detects an environmental change to a cognate response regulator (RR) that executes an adaptive action. RRs of two or more domains usually contain a structurally conserved N-terminal “receiver” domain (RD) that houses the phosphorylation site, and a C-terminal “output” domain (OD) that can have an array of functions. Phosphorylation of the RD elicits a response from the OD, the most prevalent being DNA binding and transcriptional regulation [2, 3].

**Fig. 1:**
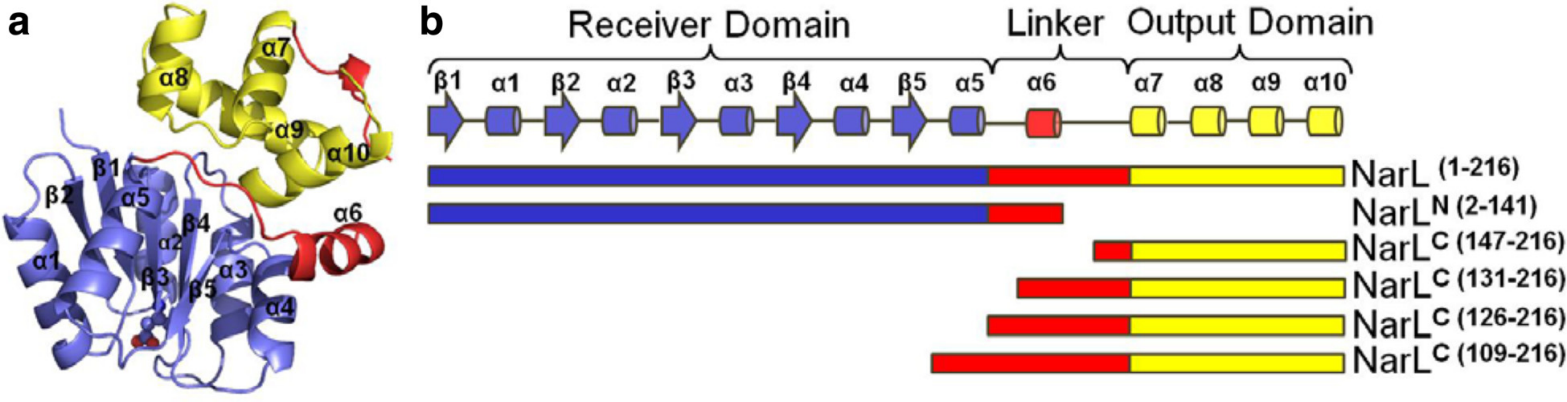
Diagrammatic representation of the NarL constructs used in this study. (**a**) Ribbon representation of NarL (PDB ID: 1A04), created using Pymol [51]. The RD is colored blue, the linker region in red, and the OD in yellow. The interdomain interface masks the DNA binding elements of the α9 recognition helix, while the linker blocks the α10 dimerization helix. The site of phosphorylation, Asp59, is represented by molecular spheres. The region between helices α6-α7 was too disordered to be resolved. (**b**) The constructs used in this study are designated by colored bars following the same color scheme as in (a). Residue range is indicated, as is the corresponding secondary structure. Arrows in the secondary structure diagram represent β-strands while cylinders represent α-helices

In *Escherichia coli*, the availability of nitrate during anaerobiosis triggers the Nar two-component system [4, 5]. Phosphoryl transfer occurs from dual sensor kinases NarX and NarQ to the two RRs NarL and NarP. NarL and NarP are transcription factors, which together regulate a family of genes involved in anaerobic respiration and fermentation [6]. Structures of full-length, unphosphorylated NarL [7, 8] from *E. coli* depict a canonical N-terminal RD, a DNA-binding C-terminal OD, and a 32-residue linker that joins them (Fig. 1a). In the unphosphorylated conformation, the RD occludes the DNA recognition helix (α9) of the DNA binding OD [7]. The activation mechanism implicit in the structure, whereby phosphorylation serves to disrupt the interdomain interface and to liberate the OD, was also corroborated by biochemical studies [9–11]. Once liberated, the NarL OD binds consensus sequences along promoter regions, with preference for inverted DNA heptamers separated by two base-pairs, called 7-2-7 sites [12]. Structures of the isolated NarL OD bound to DNA [11, 13] revealed that the OD binds 7-2-7 sites as a dimer, whereby dimerization occurs along helix α10. This finding expands the activation mechanism to include the movement of linker-helix α6 so as to render helix α10 available for dimerization.

Most RRs are governed by their oligomeric state before and after phosphorylation, and integral to this process is the oligomeric state of the RD. In RRs that act as transcription factors, phosphorylation usually results in RD dimerization, which serves to either enhance or directly stimulate DNA-binding and, hence, transcriptional activity [14]. Consistently, precluding RD dimerization by mutagenesis compromises these functions [15–18]. Phosphorylation-induced RD dimerization occurs regardless of whether the OD is initially inhibited by the RD in the unphosphorylated state, such as in FixJ and PhoB [16, 19], or is expected to be uninhibited in the unphosphorylated state, such as in OmpR and PhoP [15, 20–23]. The difference is that isolated ODs that are otherwise inhibited in the full-length protein often have some level of intrinsic function. For example, the isolated OD of PhoB activates transcription of *phoA* and the presence of RD dimers enhances this activity [16, 24]. In contrast, the OmpR OD binds DNA weakly and is unable to activate transcription without its RD [25]. Little is known about the oligomeric state of the NarL RD after phosphorylation, nor of its role in transcriptional regulation. The NarL OD, as shown by Lin and Stewart [26], is able to confer *in vivo* transcriptional activation at some promoters in the presence of nitrate, such as the *napF* promoter, and, to a lesser extent, at the *yeaR* promoter. Thus, there has been evidence that independent function of the isolated NarL OD is variable and promoter dependent, and therefore the role of the RD may be as well. We sought to further investigate this idea by examining the ability of the NarL OD to regulate transcription at two specific promoter regions: the fumarate reductase (*frdA)* promoter where NarL acts as a repressor in the presence of nitrate, and the nitrate reductase (*narG)* promoter where NarL acts as an activator in the presence of nitrate.

In this study, we examine the roles of the NarL OD, RD, and linker region in protein function and transcriptional regulation. Protein truncations of the isolated OD and RD, containing various portions of the linker, were created. These constructs were tested for their ability to regulate the *in vivo* expression of *frdA-lacZ* or *narG-lacZ* reporter fusion genes, in the presence or absence of nitrate, as compared to the full-length protein. These assays demonstrated that the NarL OD alone was sufficient to repress *frdA-lacZ* expression, regardless of nitrate availability. Incremental additions of the linker to the OD reversed *frdA-lacZ* repression and also impaired DNA binding. At the *narG* promoter, gene activation required the activities of both the OD and RD. A footprint assay showed that optimal binding of the OD to *narG* induction sites required the phosphorylated RD, which was also shown to form dimers by analytical ultracentrifugation. The variation of independent function by the NarL OD at the two promoters leads to the conclusion that the NarL RD has promoter specific functions that can extend beyond blocking and releasing the OD.

## Results

### The NarL OD represses *frdA* expression but is unable to activate *narG* expression

**Fig. 2:**
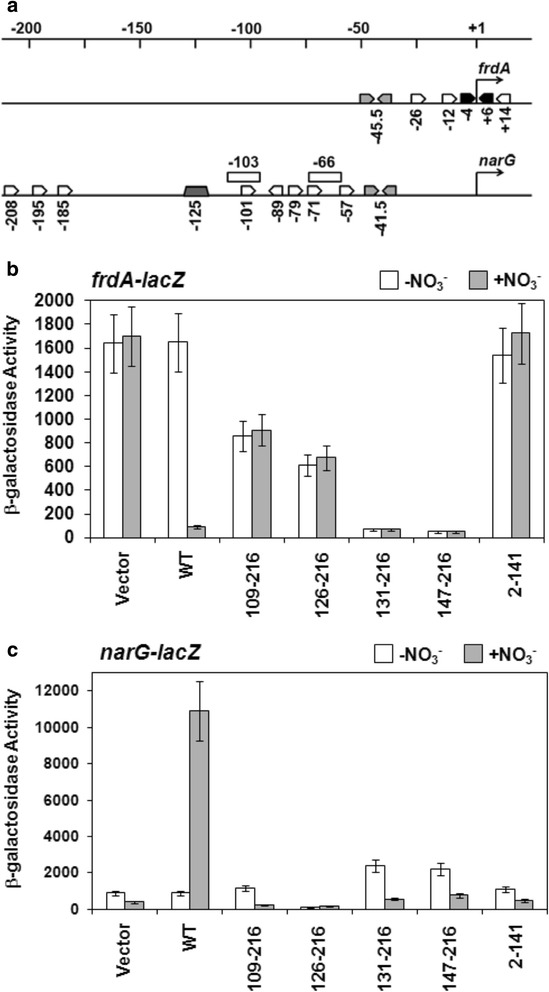
The ability of the NarL protein constructs to repress *frdA-lacZ* or activate *narG-lacZ*. (**a**) Transcription factor binding sites along the *frdA* and *narG* promoters. Black inverted arrows represent NarL high-affinity, 7-2-7 binding arrangements; white arrows represent NarL non-7-2-7 sites or NarL single heptameric sites; light gray inverted arrows represent FNR sites, a dark gray trapezoid represents the IHF binding site; white rectangular boxes represent FIS binding sites. The scale denotes nucleotides, and an arrow at the +1 nucleotide represents the transcription start site. Information used to generate this image was taken from the EcoCyc database [52] and references therein. (**b**) The ability of the NarL proteins to repress *frdA-lacZ* expression, or (**c**) to activate *narG-lacZ* expression. NarL truncated proteins were tested for anaerobic *in vivo* activity: the indicated *lacZ* transcriptional reporter fusions were used in *E. coli* MC4100 strains (ΔnarX/L/Q) harboring pACYC184 plasmid derivatives that contained NarX and either NarL^+^ or the indicated truncated NarL derivative. Levels of activity are measured in nmol OMPG hydrolyzed per min per mg protein. White bars indicate activity in the absence of nitrate, gray bars indicate activity in the presence of nitrate. Vector refers to parent plasmid pACYC184 run as a control. Error bars (vertical lines) represent one standard deviation, based on the mean value for at least three repeat experiments

In the *E. coli* cell, the presence of nitrate triggers phosphorylation of NarL by NarX. Phosphorylated NarL (NarL-P) subsequently activates genes involved in nitrate respiration and represses the expression of genes encoded for alternative respiratory pathways [4, 27]. At the *frdA* promoter for the fumarate reductase operon, NarL-P acts as a transcriptional repressor. It binds to several target DNA binding sites that are located near the transcription start site, one of which is a high**-**affinity 7-2-7 site (Fig. 2a) [28]. To determine whether the NarL OD can confer independent transcriptional regulation at this promoter *in vivo*, different NarL C-terminal domain (NarL^C^) constructs were evaluated for their ability to implement nitrate-dependent repression of an *frdA-lacZ* reporter fusion gene. The NarL^C^ constructs contained various linker lengths (Fig. 1b) , ranging from the shortest, which contained a small portion of the linker (NarL^C (147–216)^), to the longest, which contained the entire linker region (NarL^C (126–216)^). An additional construct included helix α5 of the RD (NarL^C (109–216)^). The NarL N-terminal domain plus linker region (NarL^N^) was included as a negative control. Wild-type NarL displayed a 15-fold decrease in *frdA-lacZ* promoter activity in the presence of nitrate, as opposed to the full induction of this gene in the absence of nitrate when NarL is unphosphorylated. In contrast, all NarL^C^ constructs tested at this promoter lacked a nitrate-dependent response as demonstrated by their constitutive repressive behavior (Fig. 2b). Additionally, these derivative proteins displayed two patterns of activity. Those missing the α5-α6 loop (NarL^C (147–216)^ and NarL^C (131–216)^) bestowed a level of repression comparable to that of wild-type. Those containing the α5-α6 loop (NarL^C (126–216)^ and NarL^C (109–216)^), resulted in intermediate expression; that is, their ability to repress transcription decreased by 7-fold and 10-fold, respectively, as compared to wild-type NarL. As expected, NarL^N^ was unable to repress expression since it lacked the DNA recognition domain. These results demonstrate that the NarL OD, tolerant of a certain amount of attached linker, is sufficient to repress *frdA* transcription and does so in a constitutive manner.

At the *narG* promoter for the nitrate reductase operon, NarL-P acts as a transcriptional activator. Here, the consensus DNA binding sites are located upstream from the transcription start site and lack any 7-2-7 sites (Fig. 2a). When the different NarL proteins were evaluated for their ability to activate transcription of a *narG-lacZ* reporter fusion gene *in vivo*, none of the NarL^C^ proteins stimulated transcription relative to wild-type NarL (Fig. 2c). NarL^C (147–216)^ and NarL^C (131–216)^ conferred a slight elevation of activity in the absence of nitrate when compared to the vector control, but showed the same low level expression pattern. In contrast, wild-type NarL gave rise to a 20-fold increase in activity in response to the presence of nitrate. As expected, the NarL^N^ control was unable to activate *narG-lacZ* gene expression. These results show that the RD is essential for NarL to activate the *narG* operon. Western blot analysis for these and the above transcription assays showed that all of the NarX and NarL proteins were expressed *in vivo*, and in relatively uniform concentrations (data not shown).

### The NarL RD is required for proper binding to the *narG* promoter region

**Fig. 3:**
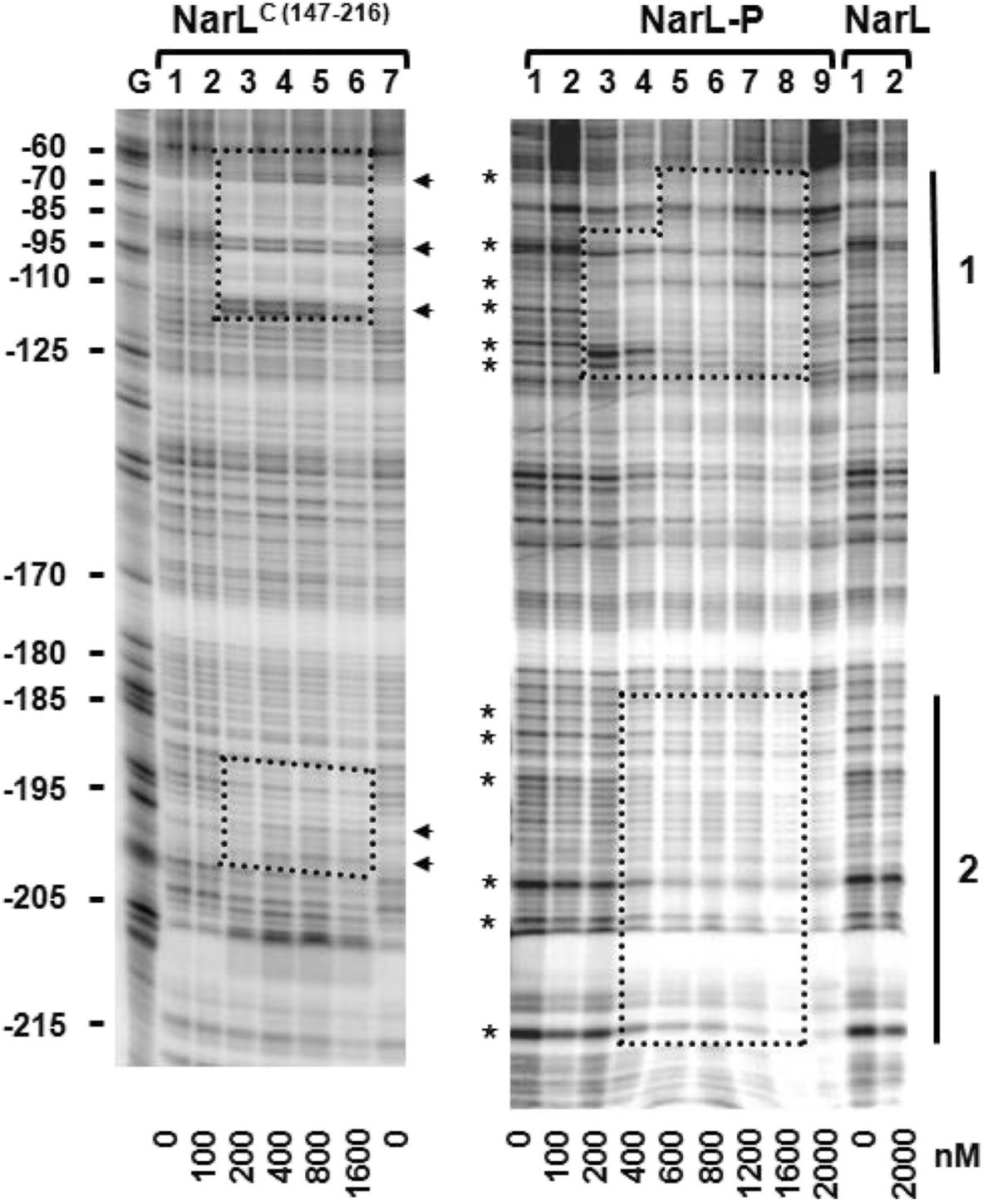
DNase I protection patterns for NarL^C (147–216)^, NarL, and NarL-P. Binding to a 391 base pair fragment containing the *narG* promoter region is shown. The vertical bars indicate the two major regions of protection seen for NarL-P, which are not as strong or as extensive in NarL^C (147–216)^. Dotted lines highlight areas of protection. Asterisks indicate altered banding patterns or hypersensitive bands for NarL-P. Arrows indicate altered banding patterns for NarL^C (147–216)^. Maxam-Gilbert G-reactions performed on the same DNA fragment were used as size markers. Coordinates relative to the transcription start site are given in base-pairs, and protein concentrations are indicated

One reason for the lack of *narG* activation by all of the NarL^C^ constructs may be due to poor binding at this promoter region. Previous DNAse I footprint analyses of the *narG* promoter has revealed two areas of protection by NarL-P: a high affinity site centered in the −89 region and a lower affinity site centered in the −195 region [28, 29]. Occupancy of both regions is necessary for full induction of *narG* transcription, in addition to other transcription factors, such as FNR and IHF that must also bind to the region [30, 31]. To determine whether the isolated NarL OD is capable of binding these critical regions, a DNAse I protection assay was performed using a 391 base-pair fragment containing the *narG* promoter region (+155 to −236). Binding patterns of NarL^C (147–216)^, NarL, and NarL-P were compared. NarL-P clearly protected both the −89 and −195 regions (Fig. 3), including regions around −150, while unphosphorylated NarL did not produce any protection pattern. NarL^C (147–216)^ conferred a strong yet different protection pattern at the −89 region, and displayed much weaker protection at the −195 region as compared to NarL-P. This weak interaction primarily resulted from alterations in hypersensitive sites and not by typical zones of protection. Although NarL-P and NarL^C (147–216)^ both bind the −89 site with the same relative affinity, their different binding pattern may be suggestive of improper occupancy by NarL^C (147–216)^. At the −195 region, NarL-P binds more extensively and with a stronger affinity. The compromised binding by the OD at the −195 region signifies at least one reason for the inability of NarL^C (147–216)^ to activate *narG-lacZ* expression. At this promoter region, the phosphorylated RD is required to impart enhanced binding affinity and full occupancy.

### Loop α5-α6 inhibits DNA binding of the NarL OD

**Fig. 4:**
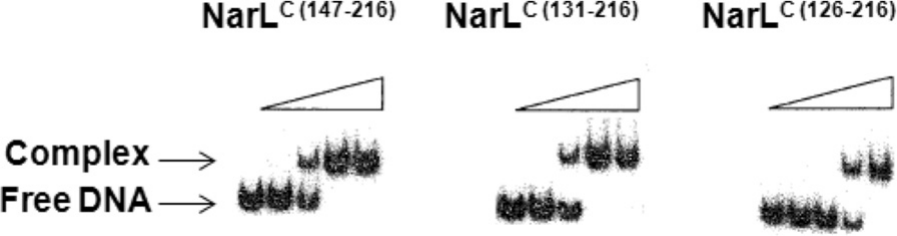
The affect of linker loop α5-α6 on NarL^C^ binding to DNA. An EMSA illustrates the relative binding affinities of the indicated NarL^C^ proteins to a ^32^P-labeled DNA fragment containing an engineered 7-2-7 *narG −*89/-89 binding site. Lanes 1–5 show increasing concentrations of the specified NarL^C^ protein (from left to right: 0, 0.25, 0.5, 1.0, and 2.0 μM). The binding affinities of NarL^C (147–216)^ and NarL^C (131–216)^, containing helix α6, are equivalent while that of NarL^C (126–216)^, containing helix α6 and loop α5-α6, is relatively reduced

The impaired *frdA-lacZ* repression by NarL^C^ constructs containing loop α5-α6 (Fig. 2b) indicates that these proteins weakly bind DNA as compared to NarL^C^ domains lacking this linker segment. To further investigate the effect of loop α5-α6 on DNA binding, we performed an EMSA using the previously constructed oligonucleotide containing the engineered *narG −*89/−89 sequence [11]. NarL^C (147–216)^, which bestows full *frdA-lacZ* repression (Fig. 2b), binds to this sequence with equivalent binding affinity as wild-type NarL-P in a DNAse I footprint assay [11], and co-crystallizes with this 7-2-7 site as a dimer [13]. Therefore, this oligonucleotide serves as a useful standard to compare the relative binding affinities of the NarL^C^ constructs used in this study. The EMSA shows that NarL^C (131–216)^, which contains helix α6, bound DNA with equivalent binding affinity as the NarL^C (147–216)^ control (Fig. 4). Consistent with the results of the *in vivofrdA-lacZ* expression assay, the presence of helix α6 did not impair DNA binding. In contrast, NarL^C (126–216)^ bound DNA less tightly, supporting the hypothesis that loop α5-α6 exerts an inhibition on the NarL OD. The same results were observed by the NarL^C^ truncation proteins when the (His)_6_-tag was placed at the C-terminal end (data not shown), suggesting that the inhibition by the α5-α6 loop is not due to the (His)_6_-tag. Furthermore, NarL^C (131–216)^ and NarL^C (126–216)^ are expected to have adopted an overall correct fold (see Discussion).

### The NarL RD dimerizes in solution while the NarL OD is monomeric

In several RRs, phosphorylation of the RD enhances DNA binding. This is often accompanied by, or a direct result of, RD dimerization [14, 15, 19, 20]. Therefore, the requirement of the phosphorylated RD to achieve optimal NarL binding at the *narG* promoter may involve RD dimerization. To determine if the NarL RD is able to dimerize and to gain further insight into the NarL phosphorylation mechanism, analytical ultracentrifugation was applied to NarL^N^ and NarL in native and phosphorylated states, and to the NarL^C^ constructs.

**Fig. 5:**
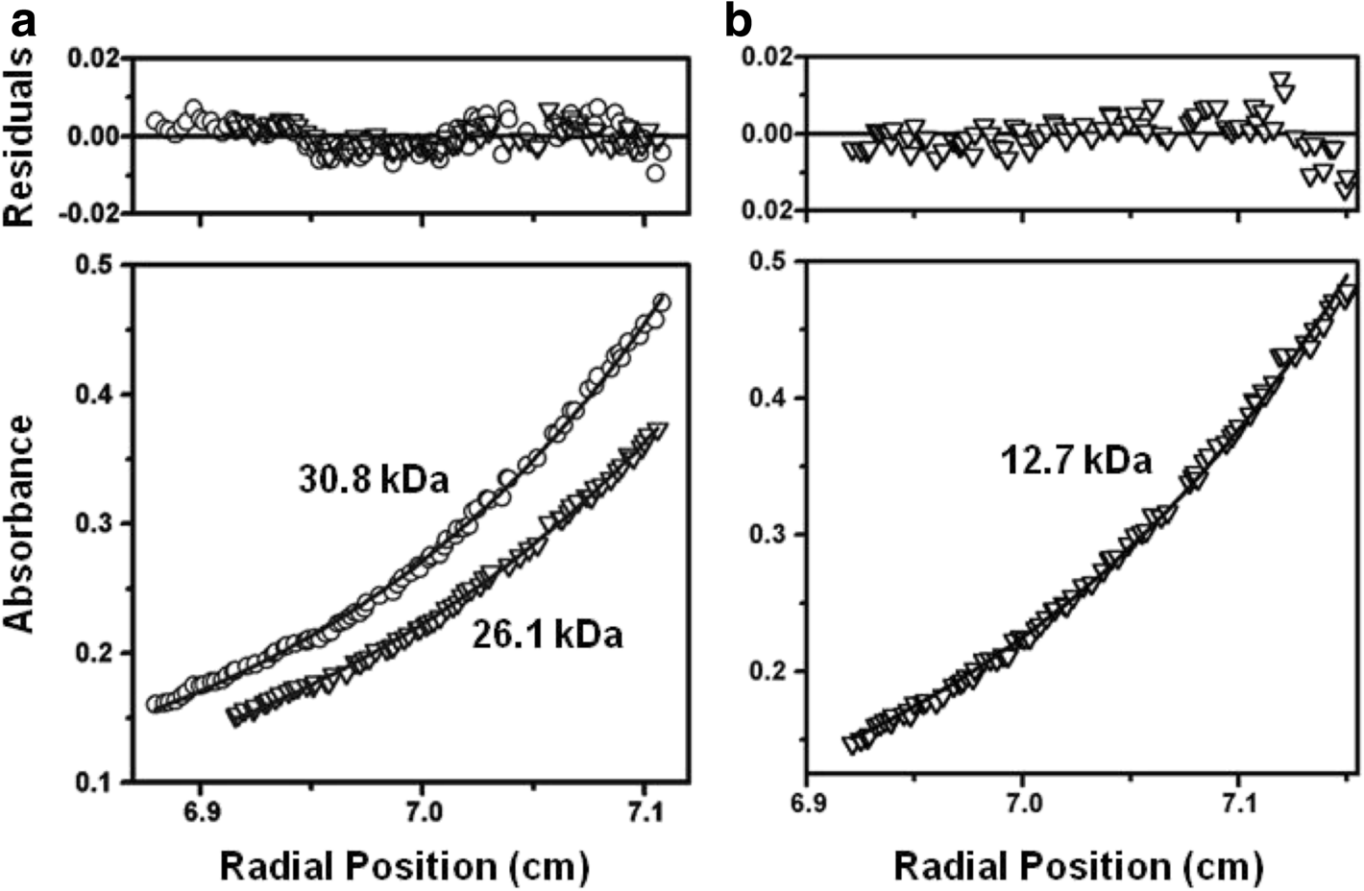
Representative sedimentation equilibrium plots. Plots are single exponential fits with the residuals shown in the upper plot of each panel. Triangles represent unphosphorylated proteins, circles represent phosphorylated proteins. Samples shown were run at a concentration of 60 μM and the measured molecular-masses in kiloDalton (kDa) are indicated. (**a**) NarL^N^ and NarL^N^-P after reaching equilibrium at 15,000 rpm and 240 nm. The sequence determined molecular-mass of NarL^N^ is 16.5 kDa, therefore NarL^N^ exists as a monomer and dimer while NarL^N^-P is mostly a dimer. (**b**) NarL^C (126–216)^, with a sequence-determined molecular mass of 11.8 kDa, after reaching equilibrium at 22,000 rpm and 280 nm. This construct, as with the shorter NarL^C (147–216)^ construct, is predominantly a monomer

NarL^N^, containing the RD and linker-helix α6, has a sequence molecular-mass of 16.5 kDa. When examined by analytical ultracentrifugation at a concentration of 60 μM, the NarL^N^ speed dependent molecular-masses were 26.1 kDa (15,000 rpm), 24.9 kDa (18,000 rpm), and 23.4 kDa (22,000 rpm) which were best fit by a monomer and dimer (Table 1 and Fig. 5a). At the corresponding centrifugation speeds, phosphorylated NarL^N^ (NarL^N^-P) gave apparent molecular masses of 30.8 kDa, 30.2 kDa and 29.0 kDa, and were best fit by a population largely in the dimeric form. When examined at 160 μM, similar results were observed and with reasonably good residuals. Unphosphorylated NarL^N^ had apparent molecular-masses of 25.9 kDa (15,000 rpm) and 23.0 kDa (22,000 rpm), while those for NarL^N^-P were 30.4 kDa (15,000 rpm) and 27.4 kDa (22,000). The slightly lower-than-expected measured molecular masses for NarL^N^-P (expected ~32 kDa) were somewhat surprising. NarL^N^-P autophosphorylates more efficiently with acetyl phosphate than full-length NarL (Additional file 1: Figure S1A), is observably more soluble, and remains phosphorylated for at least 12 hours and gradually dephosphorylates over a period of several days [10]. Although partial loss of phosphorylation in these experiments may have occurred, the observation that unphosphorylated NarL^N^ consistently displayed 50 % dimeric behavior demonstrates that this domain can form dimers when liberated from the OD. Phosphorylation then serves to increase the population of RD dimers.

**Table 1:** Summary of sedimentation equilibrium results^*a,b*^

Construct	Sequence MM^*c*^ (kDa)^*c*^	Speed (krpm)^*c*^	Measured MM at 60 μM (kDa)	Measured MM at 160 μM (kDa)	Predominant Oligomeric State
NarL^C (147–216)^	9.6	15	9.7	12.3	Monomer
		22	9.6	11.4	
NarL^C (126–216)^	11.8	15	14.1	13.6	Monomer
		22	12.7	12.4	
NarL	23.9	11	24.0	23.9	Monomer
		15	22.6	23.0	
NarL^N^	16.5	15	26.1	25.9	Monomer/Dimer
		22	23.4	23.0	
NarL^N^-P	16.6	15	30.8	30.4	Dimer
		22	29.0	27.4	

^*a*^Certain constructs had additional data collected at other speeds that are not shown in the table but were included in determining the best oligomeric fit (discussed in text)

^*b*^All runs shown were carried out in a buffer solution containing 25 mM Tris, pH 7.5-8.5 and 500 mM NaCl, except for two cases: NarL^C (147–214)^ at 60 μM contained 500 mM (NH_4_)_2_SO_4_, and NarL at 60 μM contained 100 mM NaCl. These changes to salt did not significantly alter the measured molecular masses (see Materials and Methods)

^*c*^MM: molecular mass; kDa: kiloDalton; krpm: kilorevolutions per minute

Full-length unphosphorylated NarL was monomeric at 60 μM and 160 μM, with a measured molecular-mass of 24.0 kDa at 11,000 rpm, and with small fitting residuals (Table 1). Its sequence molecular-mass is 23.9 kDa. Consistent results were also observed at the same two concentrations and at 15,000 rpm. Attempts to determine the oligomeric state of NarL-P repeatedly showed that it was dimeric, and perhaps contained a small population of tetramer. However, the results were deemed inconclusive due to the heterogeneity of the sample and the inability to rule out protein aggregation.

As mentioned, the NarL^C (147–216)^ domain co-crystallizes with a DNA 7-2-7 site as a dimer [11, 13]; but whether this truncation protein can dimerize in the absence of DNA has not been demonstrated. The molecular mass of NarL^C (147–216)^ was determined by sedimentation to be 9.6 kDa at 60 μM and 22,000 rpm, which is identical to its sequence molecular mass. This domain, therefore, remains monomeric in solution (Table 1). No salt conditions tested were found to promote dimerization, and NarL^C (147–216)^ was also predominantly monomeric at 160 μM and 22,000 rpm. To test whether the linker region affects NarL^C^ dimerization, the NarL^C (126–216)^ construct, which contains the entire linker region, was also examined by sedimentation. The molecular mass of NarL^C (126–216)^ was determined at 22,000 rpm and 60 μM to be 12.7 kDa (Table 1 and Fig. 5b) and 12.4 kDa at 160 μM. Compared to its sequence molecular-mass of 11.8 kDa, NarL^C (126–216)^ was also predominately monomeric. Residuals from the exponential fitting were small, indicating little molecular mass heterogeneity. For both NarL^C^ constructs, additional data were taken at speeds of 15,000 rpm and 18,000 rpm, which gave similar results. Taken together, these sedimentation results demonstrate that NarL^C^, either alone or with the linker attached, is predominantly monomeric in solution.

## Discussion

### The roles of the NarL RD are promoter specific

**Fig. 6:**
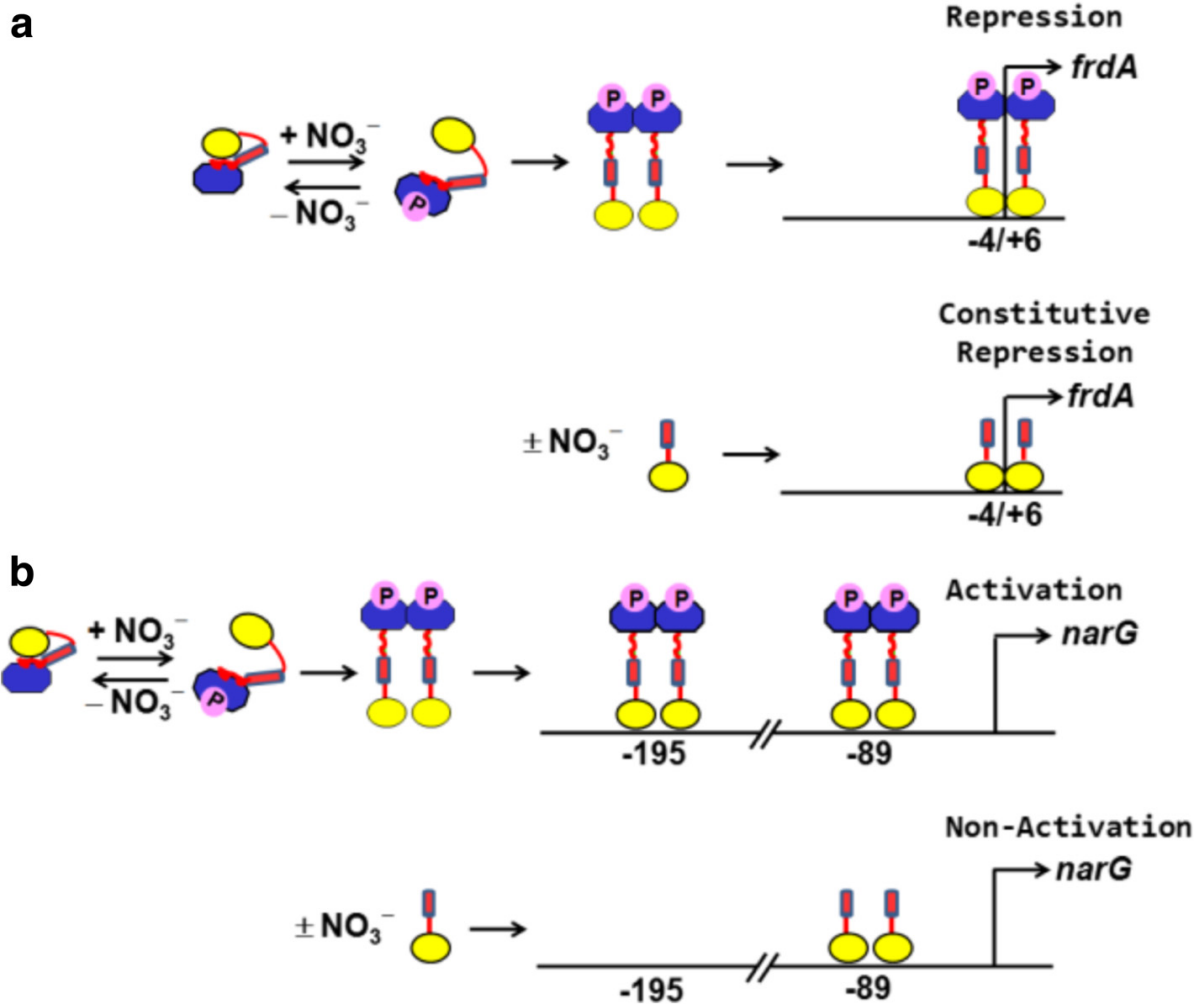
The roles of the OD and RD at the *frdA* and *narG* promoter regions. NarL is represented by a cartoon figure: the OD is depicted by a yellow circle; the linker region is in red, with the α5-α6 loop shown as a squiggle and helix α6 as a cylinder; the RD is shown as a blue irregular octagon with phosphorylation represented by a “P”. For simplicity, only preferred binding sites or regions are designated at each promoter, which are not drawn to scale. The transcription start site is indicated by an arrow. (**a**) The *frdA* promoter. Top panel: wild-type, phosphorylated, NarL binds to the *frdA* promoter region, leading to gene repression in a nitrate responsive manner. Bottom panel: NarL^C^ (which may include helix α6) is also able to repress *frdA* gene expression, but does so in a constitutive manner. The ODs are shown to dimerize at the 7-2-7 (−4/+6) binding region near the transcription start site. (**b**) The *narG* promoter. Top panel: wild-type, phosphorylated, NarL binds the essential −89 and −195 regions of the *narG* promoter to activate gene expression. The RD is presumed to dimerize and may also engage in other protein-protein interactions that are not depicted in the diagram. Bottom panel: NarL^C^ (which may include helix α6) cannot activate the *narG* promoter due to improper binding at the −195 region. Therefore *narG* operon expression remains low or non-activated in the absence of a phosphorylated RD

Our *in vivo* and *in vitro* results indicate that the NarL RD performs different functions to regulate transcription, which depend on the requirements governing specific promoter regions. This idea is summarized in Fig. 6, which depicts the roles of the OD and RD at the preferred sites of the *frdA* and *narG* promoters. Transcriptional repression of *frdA* presumably involves occlusion of RNA polymerase and the transcriptional activator FNR at the transcription start site [32, 33]. According to our results, this repression can be accomplished merely with a liberated OD, which can include linker-helix α6 (Fig. 6a). The NarL RD is not required for these functions. Instead, the role of the RD at this promoter is to prevent uncontrolled gene repression by sequestering the OD in the absence of nitrate. The RD essentially acts as a phosphorylation controlled on-off switch that transitions from the open to closed form of the protein, respectively, in response to nitrate availability. RD dimerization is proposed to occur and may do so to maintain the open form of the protein.

In contrast, the same NarL^C^ constructs that conferred strong constitutive repression of *frdA-lacZ* (NarL^C (147–216^ and NarL^C (131–216)^) were unable to activate *narG-lacZ* expression and their behavior was not constitutive. Based on our footprint experiment, the lack of *narG* activation by these constructs is at least attributed to their inability to properly bind the essential −195 region. Thus, release of the sequestered NarL OD alone is insufficient to induce transcription of the *narG* operon; rather, the presence of the NarL RD is also mandatory for transcriptional activation (Fig. 6b). At this promoter, the NarL RD is required for optimal DNA binding and occupancy, which may be facilitated by RD dimerization. Another possible role for the RD at this promoter, though not exclusive of the first, is that the NarL RD may be required to bend DNA or may be involved in cooperative interactions. Cooperativity at the *narG* promoter is thought to occur via NarL oligomerization along the DNA in conjunction with DNA bending by IHF [29, 34]. The NarL RD may be required for such functions, and possibly other protein-protein interactions. Similarly, the receiver domain of the RR TodT, a member of the NarL/FixJ family, is required to induce a hairpin bend at its promoter region that is stabilized by IHF and is required for transcription [35].

At the *narG* promoter, NarL is reminiscent of RRs with unblocked ODs in the pre-phosphorylated state, such as OmpR or UhpA, which can bind DNA (and may initiate basal transcription) but require phosphorylation to enhance binding and bestow full transcriptional activation [36–38]. In other words, sequestering the NarL OD from binding to the *narG* promoter is not as important as sequestering it from binding to the *frdA* promoter. Therefore, a response regulator having a sequestered OD does not necessarily indicate that the OD has intrinsic function at all times. Likewise, the OD of DevR (or DosR), a homologue of NarL, is also inhibited in the inactive state, but during hypoxia the DevR OD alone is unable to activate transcription of genes that require cooperativity [39]. Cooperative binding requires the DevR RD. Thus, in situations where the NarL OD has little to no intrinsic function, the NarL RD has an additional regulatory role, the extent of which varies between promoter regions.

Our results are also consistent with the aforementioned NarL studies by Lin and Stewart [26] that showed different extents of OD activity at the *napF* verses *yeaR* promoters. NarL^C^ alone was able to fully activate *napF* and only partially activate *yeaR* despite both having a 7–2–7 binding site upstream of the transcription start site (−40 to −49 for *napF* and −38 to −47 for *yeaR*). This also implies ﻿that the functions of the NarL RD depend more on promoter specificity than on the location of the NarL binding sites. Discrepant roles for the RD at different promoter regions have also been observed or implicated in other RRs. Unphosphorylated PhoP, which does not have a blocked OD, can bind DNA derived from the *phoP* promoter, but requires a phosphorylated RD to bind DNA derived from the *msl3* promoter. The RD of FixJ was proposed to be dispensable at the *nifA* promoter [40], since, when liberated, the OD was able to confer gene activation comparable to wild-type levels. However phosphorylation-induced RD dimerization of FixJ significantly raised the binding affinity of FixJ to *fixK* and is expected to be vital at this promoter [19].

### Helix α6 verses loop α5-α6

We investigated the affects of specific linker segments on NarL OD function and found discrepant activities between OD constructs containing linker helix α6 verses those containing linker loop α5-α6. The presence of helix α6, in NarL^C (131–216)^, bestowed similar behavior to that seen in NarL^C (147–216)^, which lacks this helix and is known to fold correctly from its crystal structure [11]. Since both NarL^C (131–216)^ and NarL^C (147–216)^ showed constitutive repression of *frdA-lacZ* expression and similar DNA binding *in vitro*, this indicates that NarL^C (131–216)^ also folded correctly. Supporting evidence that NarL^C (131–216)^ (and NarL^C (126–216)^) folded correctly stems from the NMR structure of the *Erwinia amylovora* RcsB OD and linker segment, which shows structural homology to the corresponding region (residues 129–216) in full-length NarL [41]. Therefore, helix α6 in NarL^C (131–216)^ is predicted to form hydrophobic contacts with helix α10, which, in this form, blocks OD dimerization. These modest interactions, however, could render helix α6 flexible and account for its apparent movement that enabled NarL^C (131–216)^ to function properly. Thus, our data support the expected relocation of helix α6 upon NarL phosphorylation that would enable OD dimerization on DNA via helix α10 [11].

**Fig. 7:**
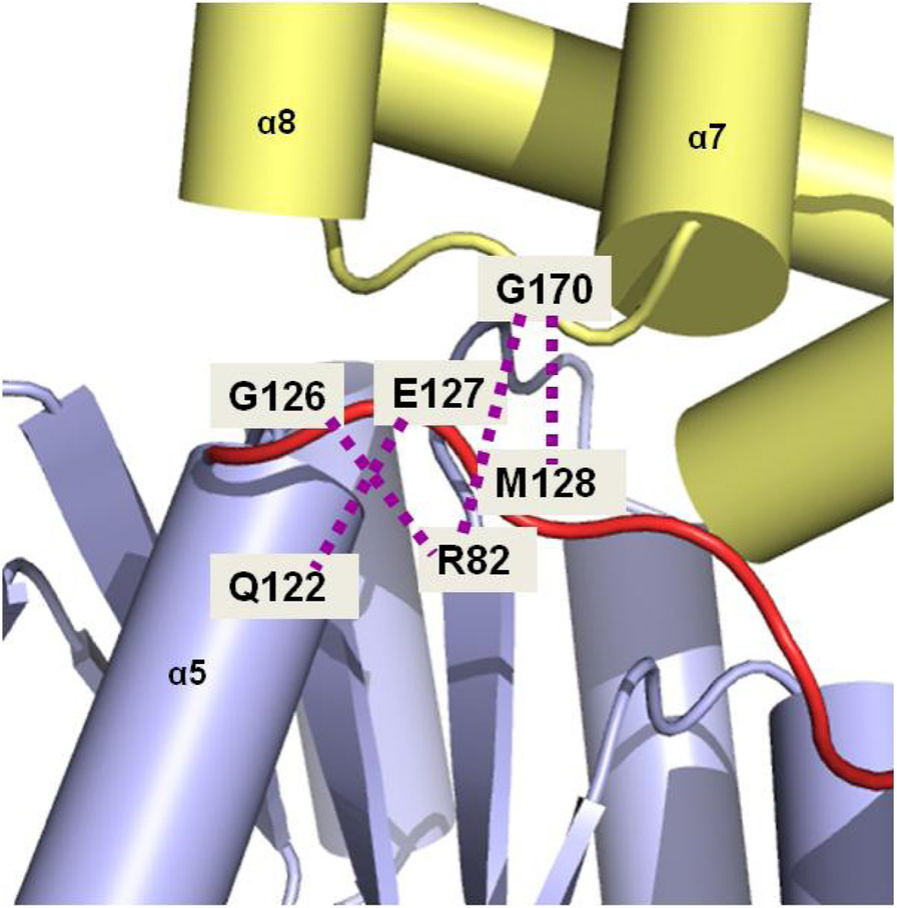
Hydrogen bond contacts in the region of loop α5-α6. Residues 126–128 within the α5-α6 loop (red) form hydrogen bond contacts (purple dotted lines) with both the RD (light blue) and OD (light yellow) of NarL. An interdomain contact between R82 and G170 is also depicted. Removal of the RD abolishes the hydrogen bonds in this region except that of M128 and G170

In contrast, the addition of loop α5-α6, such as in NarL^C (126–216)^, compromised *frdA-lacZ* repression *in vivo* and weakened DNA binding to a high affinity 7–2–7 site *in vitro*. This may be explained by the hydrogen bond between M128 in linker loop α5-α6 and G170 in loop α7-α8 of the OD (Fig. 7), which is the only remaining contact for loop α5-α6 once the RD is removed. This hydrogen bond, presumably present in NarL^C (126–216)^, may partially anchor the OD in this region and impart the relative intermediate inhibition observed. By this activation model, release of the OD upon NarL phosphorylation entails severing contacts between the OD and loop α5-α6. This activation mechanism also correlates with our previous EPR studies whereby phosphorylation was proposed to induce a hinge bending movement in the vicinity of the Gly126 [9]. Interestingly, linker involvement at the domain interface and activation mechanism has also been shown in other RRs. In CheB, the linker contributes to the interface and mutations to it can result in methylesterase activity that bypasses phosphorylation [42]. Helix α6 of full-length VraR (which is equivalent to helix α6 in NarL) is thought to stabilize the interdomain interface, and activation by beryllofluoride unwinds this helix as part of releasing the OD [17]. Likewise is observed for the full-length structure of Spr1814 in *S. pneumonia* where the presumed semi-activated form shows a conformational change of the same helix α6 and the loop which follows [43].

## Conclusions

Based on our results, we propose that phosphorylation of NarL has a dual purpose, to relieve OD inhibition and to enable RD homodimers. Liberation of the OD would entail severing contacts between the RD and OD as wells as between the linker and OD. Once separated, the domains play independent, yet concerted, roles in regulating gene expression. Liberation of the OD alone is sufficient to control some promoters, while control of other promoters requires additional events involving the receiver domain. Thus, the RD can act as a molecular switch or bestow additional complex functions, such as increasing the DNA binding affinity and forming other protein-protein interactions, which render it completely necessary for stimulating transcription.

## Methods

### Bacterial strains, plasmids, and bacteriophages

The bacterial strains, bacteriophages, and plasmids used in this study are listed in Table 2. All bacterial strains are derived from MC4100 F^−^*araD139* Δ(*argF-lac)U169 rpsLl50 relA1 flb-5301 deoCl ptsF25 rbsR* [44]. Desired constructs were confirmed with DNA sequence analysis and subsequent subcloning of all pLK63 plasmid derivatives were constructed as previously described [45]. Bacterial strain JM109 and plasmid pQE9 (Qiagen) were used for all protein expression experiments. For protein production and *in vitro* studies the *narL* and truncated *narL* genes were amplified from existing plasmid constructions using PCR technology. TaKaRa Extaq polymerase (Takara Holdings, Inc.), was used for PCR and the amplified products were cloned into the *Bam*Hl and *Hin*dlll restriction sites of the pQE9 plasmid. This construction adds an N-terminal histidine epitope to the NarL protein and also results in substitution of Met1 to Gly. NarL is in-frame beginning at Ser2 with the *Hin*dlll restriction site introduced 54 bases downstream from the stop codon of the *narL* coding sequence. The N-terminal NarL derivative is in-frame beginning at Ser2 with a stop codon introduced at Leu142, immediately followed by a *Hin*dIII restriction site. This construct (2–141) is referred to as “NarL^N^.” The NarL C-terminal expression constructs (referred to as “NarL^C^”) are in-frame beginning at their indicated residue with the *Hin*dIII restriction site introduced 54 bases downstream from the stop codon of the *narL* coding sequence. NarL^C^ truncation proteins were generated by PCR amplification. The amplified fragments were digested with *Bam*HI and *Hin*dIII and then subcloned into the same sites of PQE9 (or digested with *NcoI* and *Bgl*II, and subcloned into the same sites of the PQE60 vector (Qiagen) to add a C-terminal histidine epitope for use in the EMSA experiment only). All restriction endonucleases, polymerases, and other enzymes were purchased from New England Biolabs except where noted.

**Table 2:** Strains, plasmids, and phages used in this study

Strains	Parent	Genotype	Source
CJ236		F’ cat (pCJ105;M13^S^Cm^R^)	[53]
		*dut ung thi relA spoT1 mcrA*	
JM109		*endA1 recA1 gyrA96 hsdR17*	[54]
		*(r*_*k*_^*−*^ *m*_*k*_^*+*^ *) relA1supE44 thi*	
		[F^−^ *traD36 proAB lacI* ^*q*^ ZΔM15] Δ(*lac-proAB*)	
MC4100		F^−^ Δ(*argF-lac*) *U169 araD139*	[44]
		*deoC1 relA1 flbB5301 rbsR rpsL50 ptsF25*	
MV1190		Δ(*srl-recA*)306:Tn10 (tet^R^)	BioRad
		Δ*(lac-proAB*) *thi supE*	
		[F: *traD36 proAB lacI* ^*q*^ZΔM15]	
RC11	MC4100	Δ*narXL* Δ*narQ*::*kan recA56 srl*::*Tn10 tet*	[46]
Phage	Parent	Genotype	Source
M13MK1	M13mp18	EcoRI-BamHl '*narX narL* ^+^	[46]
λLK1	λRS45	φ(*frdA-lacZ*) (*hyb*) *lacY* ^*+*^ *lacA* ^*+*^	[55]
λPC51	λRS45	φ(*narG-lacZ*) (*hyb*) *lacY* ^*+*^ *IacA* ^*+*^	[56]
Plasmid	Parent	Genotype	Source
pACYC184		Cm^R^ Tc^R^	[47]
pLK63	pACYC184	*narX* ^*+*^ *narL* ^*+*^ *Cm* ^*R*^	[45]
pMJ145	pLK63	*narX* ^*+*^ *narL* ^*C (147–216)*^	This study
pMJ146	pLK63	*narX* ^*+*^ *narL* ^*C (131–216)*^	This study
pMJ152	pLK63	*narX* ^*+*^ *narL* ^*C (126–216)*^	This study
pMJ153	pLK63	*narX* ^*+*^ *narL* ^*C (109–216)*^	This study
pMJ154	pLK63	*narX* ^*+*^ *narL* ^*N (2–141)*^	This study
pQE9		Ap^R^	Qiagen
pMJ05	pQE9	*narL* ^*C (147–216)*^	This study
pMJ99	pQE9	*narL* ^*N (2–141)*^	This study
pMJ104	pQE9	*narL*	This study

### Protein expression and purification

*E. coli* JM109 cells containing the recombinant over-expression vectors were cultured in 10 mL L Broth with 100 μg/mL ampicillin overnight at 37 °C, and then transferred to 500 mL L Broth with 100 μg/mL ampicillin and grown at 37 °C until the OD_600_ reached 0.7–1.0. IPTG (Isopropyl-β-D-thiogalactopyranoside) was added to a final concentration of 2 mM. After 3 hours, the cells were harvested by centrifugation at 4000 rpm at 4 °C and then resuspended in buffer (30 mM Tris, pH 8.0, 150 mM NaCl, 2 mM MgCl_2_), with the addition of DNase (5 mg/mL) and RNase A (10 mg/mL). Cells were broken by passage through a French Press at 12,000 psi. The cell lysate was spun at 16,000 g for 10 min and the supernatant was either incubated with Qiagen Ni-nitrilotriacetic acid resin before loading onto a column, or was loaded onto a 1 mL HiTrap™ chelating Sepharose™ HP column (GE Healthcare). (His)_6_-tagged NarL proteins were purified according to the manufacturer’s instructions and the proteins were eluted with the above (or related) buffer containing 0.5 M Imidazole. Collected protein fractions were dialyzed and concentrated against a storage buffer (usually 30 mM Tris, pH 7.6, 150 mM KCl, 0.5 mM MgCl_2_, and 10 % glycerol) using an Amicon Centricon 10, and stored at −80 °C. Protein concentrations were determined using a Bio-Rad protein assay kit with BSA as the protein standard.

### β-galactosidase assays

Strain RC11 was lysogenized with either λLK1 (*frdA-lacZ*) or λPC51 (*narG-lacZ*) reporter fusions and is used in all *in vivo* β-galactosidase experiments as previously described [46]. Plasmids containing the NarL constructs, pLK63, are derived from the parent plasmid, pACYC184 (Table 2), which has been shown to exist in low cellular concentration (10–15 copies per cell) [47]. Values reported represent the averages of at least three experiments, and a standard deviation of less than 15 %.

### DNAse I footprinting assay

DNase I footprinting assays were performed essentially as described [48]. A 391 base-pair *Eco*RI-*Bam*HI fragment derived from pIS35 was used as DNA template for the footprinting assays [31]. This fragment contains the *narG* promoter region extending from nucleotides −236 to +155 relative to the start of transcription. The fragment was isolated and end-labeled by fill-in reactions using Klenow DNA polymerase fragment and [^32^P]-dATP (3000 Ci/mmole). Purified NarL or NarL^C (147–216)^ (0–2000 nM) and labeled DNA template (1.4 nM) were incubated in 20 μL complex formation buffer (10 mM Tris, pH 7.6, 50 mM KCl, 7.5 mM MgCl_2_, 3 mM CaCl_2_, 100 μg/mL BSA) for 30 min at 22 °C. For assays using phosphorylated NarL, the protein was first phosphorylated for 20 min (50 mM HEPES, pH 8.0, 50 mM KCl, 5 mM MgCl_2_, 2 mM dithiothreitol, 25 mM acetyl phosphate) and 25 mM acetyl phosphate was also added to the individual reaction mixtures. DNase I (500 pg, GE Healthcare) was then added to each reaction mixture for 30 s. The digestion reaction was terminated with the addition of 15 μL stop solution (34 mM EDTA, 6.5 M ammonium acetate). Samples were precipitated, washed with 70 % ethanol, and resusupended in 5 μL sample loading buffer (90 % formamide, 1xTBE, 0.05 % bromophenol blue, 0.05 % xylene cyanol) before separating on 8 M urea, 6 % polyacrylamide electrophoresis gels. The G sequencing reaction ladders were created using an alternate protocol to the Maxim-Gilbert sequencing reaction [48]. Gel exposure, development, and quantitation were performed using Molecular Dynamics PhosphorImager 445 SI and accompanying software.

### Electrophoretic-mobility shift assay (EMSA)

Products from the PCR reaction, using the pIS69 [11] plasmid as a template against OSB5 [11] and OSB6 [11] primers, were digested with *Eco*RI. The resulting 183 base-pair oligonucleotide contained the engineered, 7−2−7 palindromic *narG −*89/−89 site, 5’-TACCCCTAAAGGGGTA-3’ (heptamer sites underlined). The oligonucleotide was extracted from gels and labeled with ^32^P using the Klenow fragment of DNA polymerase (New England Biolabs) and purified using a Qiagen PCR clean-up kit. The EMSA was performed, as previously described [13], by incubating the ^32^P-labeled DNA probes with NarL protein constructs for 10 min at room temperature. The reaction mixtures were immediately run on a 6 % non-denaturing polyacrylamide gel electrophoresis (Invitrogen) and visualized by autoradiography.

### Sample preparation for sedimentation equilibrium experiments

Phosphorylated NarL (NarL-P) or phosphorylated NarL^N^ (NarL^N^-P) was prepared by incubating 246 μM protein with phosphorylation buffer (40 mM KCl, 50 mM Tris, pH 7.5, 40 mM MgCl_2_, 2.5 % glycerol) and acetyl phosphate (at a 1200:1 molar ratio of acetyl phosphate to protein) for 10 min at room temperature. Since NarL^N^ and NarL^N^–P are indistinguishable by gel electrophoresis, our method of phosphorylating NarL^N^ was validated using radioactive acetyl-phosphate, which showed that NarL^N^ phosphorylates more efficiently than NarL (Additional file 1: Figure S1A) and is expected to be maintained for up to several days [10]. The phosphorylation level of NarL-P used for sedimentation equilibrium was routinely evaluated on a 20 % native polyacrylamide gel electrophoresis (2 μg loaded per well) using a Phast Gel System (GE Healthcare), and revealed NarL-P yields above 70 % (Additional file 1: Figure S1B) as measured by densitometry (AlphaImager densitometer, Alpha Innotech Corp.). Small-scale electrophoretic studies of NarL-P demonstrated its relative stability for up to one week at 4 °C (data not shown). All samples used for sedimentation equilibrium were prepared fresh for each run, placed in microdialysis buttons (Hampton) using a 1,000 molecular-mass-cutoff membrane (Spectrum), and dialyzed against several changes of buffer (25 mM Tris, pH 7.5 and 500 mM NaCl). To avoid precipitation of NarL-P, dialysis began at 750 mM NaCl. Samples were then diluted to 60 μM or 160 μM. NarL^C (147–216)^ at 60 μM, however, was dialyzed in the buffer solution containing 150 mM NaCl or 500 mM (NH_4_)_2_SO_4_. Both samples were tested and showed that different salt concentrations produced no significant difference in their sedimented molecular masses. Similarly, dialyzing NarL–P in the buffer solution containing 100 mM NaCl or 500 mM NaCl did not significantly change the results.

### Sedimentation equilibrium

Sedimentation equilibrium runs were performed at 4 °C in a Beckman Optima XL-A analytical ultracentrifuge using absorption optics. Samples were examined in 3 mm double sector, 12 mm double sector and 12 mm six sector cells at an appropriate wavelength (240, 280, or 295 nm) to ensure the absorbance was sufficient to give a good signal-to-noise ratio and the maximum absorbance was within the linear range of the instrument (less than 1.35 OD). Sedimentation equilibrium profiles were measured at 11,000, 15,000 and 18,000 rpm for NarL and NarL–P and 15000, 18,000 and 22,000 rpm for NarL^C (147–216)^, NarL^C (126–216)^, NarL^N^ and NarL^N^-P. For low wavelength (240 nm) scans, a baseline was determined by pelleting the protein at 50,000 rpm. The data were initially fit with nonlinear, least-squares exponential for a single ideal species using the Beckman Origin-based software (Version 3.01) to give a weight-average molecular mass of all species in solution. When concentration and speed dependence of the molecular masses indicated association behavior, multiple runs (at least 4, including two concentrations and two different speeds) were analyzed using the “multifit” option of the Beckman global analysis software. The monomeric sequence molecular mass and various models (monomer-dimer, monomer-tetramer, and so on) were tested to see which would give the best fit to the data. Partial specific volumes of 0.740 for NarL, 0.733 for NarL^N^, 0.734 for NarL^C (147–216)^ and 0.739 for NarL^C (126–216)^ calculated from the amino acid composition and corrected to 4 °C were used [49, 50]. The calculated effect of the phosphate group on the partial specific volume was negligible, and thus ignored.

## Additional file

Additional file 1: Figure S1. NarL and NarL^N^ phosphorylation reactions. (A) The phosphorylation time course for full length NarL and NarL^N^. Each protein (25 μM) was incubated with radiolabeled acetyl phosphate (25 mM) at room temperature. Samples of NarL-P (solid line) or NarL^N^-P (dotted line) were taken at the indicated time points. Units are expressed as PhosphoImager (PI). (B) NarL phosphorylation reactions. NarL (246 μM) was phosphorylated with different ratios of acetyl phosphate (AP) at room temperature and run on the depicted 20 % native Phast gel; each lane contains ~2 μg. Lane 1- NarL only; Lane 2- NarL-P at 400:1 AP: NarL; Lane 3- NarL-P at 600:1 AP: NarL; Lane 4- NarL-P at 800:1 AP:NarL; Lane 5- NarL-P at 1200:1 AP:NarL; Lane 6- NarL-P at 400:1 AP:NarL plus 175 mM additional KCl. Lanes 5 and 6 are 76.7 and 80 % phosphorylated, respectively, as determined from an AlphaImager densitometer. Lane 5 represents the reaction used the sedimentation equilibrium experiments; lane 6 represents an improved version that was also used in the experiments. (DOCX 58 kb)

## Abbreviations

EMSA: Electrophoretic mobility shift assay
EPR: Electroparamagnetic resonance
FIS: Factor for inversion stimulation
FNR: Regulator of fumarate and nitrate reduction
IHF: Integration host factor
NMR: Nuclear magnetic resonance
OD: Output domain
RD: Receiver domain
RR: Response regulator
NarL^N^: NarL receiver domain with portion of the linker
NarL^C^: NarL output domain with portion(s) of the linker
NarL^N^-P: Phosphorylated NarL receiver domain with portion of the linker
NarL-P: Phosphorylated full length NarL protein
