# Supplementary material for: The Escherichia coli NarL receiver domain regulates transcription through promoter specific functions
Source: BMC Microbiol. 2015 Aug 26;15:174. doi: 10.1186/s12866-015-0502-9 (PMC4549865; doi:10.1186/s12866-015-0502-9)
Supplement: Additional file 1: Figure S1. — NarL and NarLN phosphorylation reactions. (A) The phosphorylation time course for full length NarL and NarLN. Each protein (25 μM) was incubated with radiolabeled acetyl phosphate (25 mM) at room temperature. Samples of NarL-P (solid line) or NarLN-P (dotted line) were taken at the indicated time points. Units are expressed as PhosphoImager (PI). (B) NarL phosphorylation reactions. NarL (246 μM) was phosphorylated with different ratios of acetyl phosphate (AP) at room temperature and run on the depicted 20 % native Phast gel; each lane contains ~2 μg. Lane 1- NarL only; Lane 2- NarL-P at 400:1 AP: NarL; Lane 3- NarL-P at 600:1 AP: NarL; Lane 4- NarL-P at 800:1 AP:NarL; Lane 5- NarL-P at 1200:1 AP:NarL; Lane 6- NarL-P at 400:1 AP:NarL plus 175 mM additional KCl. Lanes 5 and 6 are 76.7 and 80 % phosphorylated, respectively, as determined from an AlphaImager densitometer. Lane 5 represents the reaction used the sedimentation equilibrium experiments; lane 6 represents an improved version that was also used in the experiments. (DOCX 58 kb) [file 12866_2015_502_MOESM1_ESM.docx]

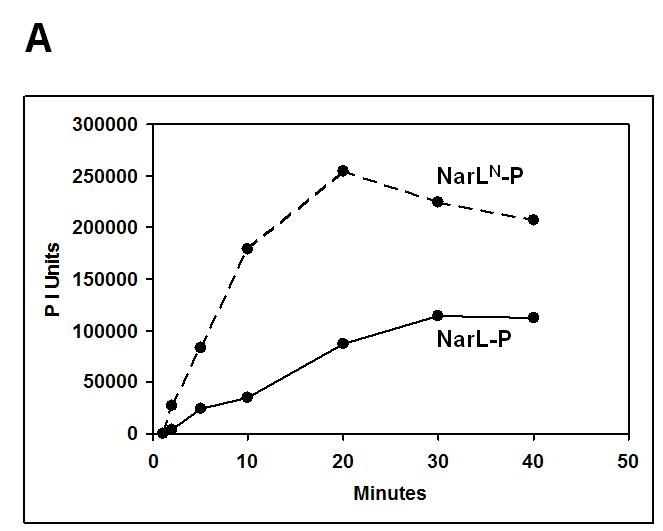


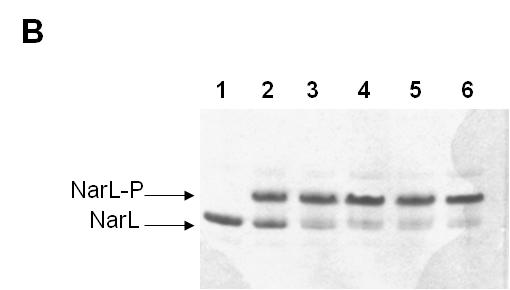


**Additional file 1: Figure S1. NarL and NarL^N^ phosphorylation reactions.**

**(A)** The phosphorylation time course for full length NarL and NarL^N^. Each protein (25 μM) was incubated with radiolabeled acetyl phosphate (25 mM) at room temperature. Samples of NarL-P (solid line) or NarL^N^-P (dotted line) were taken at the indicated time points. Units are expressed as PhosphoImager (PI). **(B)** NarL phosphorylation reactions. NarL (246 μM) was phosphorylated with different ratios of acetyl phosphate (AP) at room temperature and run on the depicted 20% native Phast gel; each lane contains ~2 μg. Lane 1- NarL only; Lane 2- NarL-P at 400:1 AP:NarL; Lane 3- NarL-P at 600:1 AP:NarL; Lane 4- NarL-P at 800:1 AP:NarL; Lane 5- NarL-P at 1200:1 AP:NarL; Lane 6- NarL-P at 400:1 AP:NarL plus 175mM additional KCl. Lanes 5 and 6 are 76.7% and 80% phosphorylated, respectively, as determined from an AlphaImager densitometer. Lane 5 represents the reaction used the sedimentation equilibrium experiments; lane 6 represents an improved version that was also used in the experiments.
